# WeChat-Based Nursing Interventions in Women’s Mobile Health: Systematic Review

**DOI:** 10.2196/82679

**Published:** 2026-07-06

**Authors:** Jun Xiao, Rui Li, Yangyang Wu, Tong Wu

**Affiliations:** 1 Department of Thyroid and Breast Surgery, Tongji Hospital, Tongji Medical College, Huazhong University of Science and Technology, Wuhan, China; 2 Emergency Department, Tongji Hospital, Tongji Medical College, Huazhong University of Science and Technology, Wuhan, China; 3 Department of Animal Science and Technology, Sichuan Agricultural University, Chengdu, China; 4 Key Laboratory of Cancer Invasion and Metastasis (Ministry of Education), Tongji Hospital, Tongji Medical College, Huazhong University of Science and Technology, Wuhan, China; 5 Department of Obstetrics and Gynecology, Tongji Hospital, Tongji Medical College, Huazhong University of Science and Technology, No. 1095, Jiefang Avenue, Wuhan, 430030, China, 86 13018044651

**Keywords:** WeChat, mobile health, mHealth, bibliometric, applet, women

## Abstract

**Background:**

Mobile health (mHealth) technology offers new approaches to improve women’s health by providing personalized monitoring and real-time guidance. As one of the most widely used social media platforms in China, WeChat has shown great potential in mHealth practice, yet systematic evidence on its application in women’s health care remains insufficient.

**Objective:**

This study aims to systematically review WeChat-based nursing interventions in women’s mHealth in order to clarify the application status, intervention modalities, target populations, and effectiveness outcomes.

**Methods:**

Searches were conducted in IEEE Xplore, Web of Science, PubMed, Scopus, ACM Digital Library, and Cochrane Central Register of Controlled Trials between January 2011 and December 2024. Two independent reviewers screened studies; extracted data on study design, intervention forms, target diseases, and outcome indicators; and assessed methodological quality. The Cohen κ coefficient was used to evaluate interreviewer agreement. Publication trends, institutional collaborations, author contributions, and research hotspots were analyzed using VOSviewer (Leiden University) and InCites (Clarivate) for bibliometric analysis.

**Results:**

A total of 31 eligible studies published from 2014 to 2024 were included. Most studies were randomized controlled trials (n=27). Intervention modalities mainly included WeChat groups (n=22), official accounts (n=18), applets (n=4), and private chats (n=9), mostly used in combination. The top focused health issues were prenatal care (n=5), breast cancer (n=5), gynecological cancer (n=5), and gestational diabetes mellitus (n=4). Six studies adopted multidisciplinary teams. Cohen κ was 0.71, indicating substantial agreement. Publications grew rapidly after 2018, peaking in 2021 and 2024. A total of 40 institutions participated, with Xi’an Jiaotong University having the highest citation impact. Most studies were at high risk of bias due to a nonblinding design.

**Conclusions:**

WeChat-based nursing interventions improve personalized health information access, self-management ability, treatment compliance, and real-time doctor-patient communication for women. This is the first systematic review to evaluate WeChat mHealth interventions in women’s health, filling the research gap. Future research should focus on improving methodological quality, exploring cross-cultural adaptability, conducting long-term follow-up, and integrating wearable devices and electronic health records to further optimize WeChat-based women’s health services.

## Introduction

Women face unique health issues at different life stages [1]. In adolescence, the onset of menstruation may bring challenges like dysmenorrhea or polycystic ovary syndrome [2], while reproductive years often involve fertility management, prenatal care, and pregnancy-related conditions [3]. During midlife, menopause introduces hormonal fluctuations that can trigger hot flashes, osteoporosis, and mood disorders [4]. Even beyond menopause, postreproductive years confer increased risks of cardiovascular disease, breast cancer, and cognitive decline [5,6]. Furthermore, women tend to be more vulnerable to anxiety and depression than men, owing to a mix of biological, psychological, and social elements [7]. Therefore, safeguarding women’s health is an important task in the public health field. It not only concerns their own quality of life but also correlates with the overall development of society.

With the quick development of information technology, mobile health (mHealth) offers a new avenue for improving women’s health. It refers to leveraging mobile devices, like smartphones and wearables, to provide accessible and tailored health care services [8]. For instance, women may use the Oura ring to track their sleep, temperature, and heart rate during the menstrual cycle, which helps find early health warning signs [9]. A high usage of the SmartMoms app in pregnant women exerts a positive effect on moderate to vigorous physical activity. Additionally, mHealth tools can provide nutrition guidance and exercise advice for pregnant and postmenopausal women [10]. Some may even promote screening attendance and promote timely preventive health care behaviors [11]. Overall, mHealth is transforming women’s health care by supporting their varied needs and making health interventions more accessible and effective.

In China, WeChat stands out as a major social media platform with a substantial user population. According to Tencent’s 2025 annual earnings report [12], WeChat had more than 1.3 billion monthly active users as of March 2025, covering most of China’s internet users. This phenomenon renders the WeChat platform as an effective mHealth platform for women’s health [13,14], with several key advantages. First, its large user base allows accurate delivery of health information and services. Second, users can share health care information online [15,16]. Third, applets can be developed into practical health management tools [17]. However, despite its convenience, it still lacks systematic reviews on the WeChat app in women’s health care.

Previous reviews have evaluated the effects of mHealth interventions on women’s health outcomes, but they did not distinguish between different mHealth delivery modalities [18,19]. In particular, WeChat-based interventions were not separately analyzed in prior syntheses. Meanwhile, existing reviews that involve WeChat have centered on chronic conditions such as cancer [20], mental health [21,22], and diabetes [23], without focusing on the full life cycle of women’s health. Under this context, it is essential to systematically explore the application, effectiveness, and implementation of WeChat-based mHealth interventions in women’s health to fill this important evidence gap.

This systematic review aims to comprehensively and systematically collect and analyze relevant research on women’s mHealth apps based on the WeChat platform, summarizing their application status, effectiveness, and existing problems. By comprehensively evaluating WeChat’s apps in all aspects of women’s health monitoring, disease prevention, and health management, it provides a scientific basis for further optimizing the application of WeChat in women’s mHealth. At the same time, this study helps to identify research gaps and development trends in this field, providing directions for subsequent research and practice. Thus, it can better use the WeChat platform to improve women’s health levels, filling the gaps in existing research, and has important theoretical and practical significance.

## Methods

### Study Design

This review was registered in the PROSPERO International Prospective Register of Systematic Reviews (CRD420251051789) and followed the PRISMA (Preferred Reporting Items for Systematic Reviews and Meta-Analyses) 2020 guidelines closely in its conduct and reporting (Checklist 1) [24].

### Research Question

This systematic review aimed to explore the research question: How effective are WeChat-based nursing interventions in enhancing health outcomes for women at various life stages, and what is their current implementation status? The design of the review adhered to the PICO framework:

1. Population (P): Women of all ages and life stages (adolescence, reproductive years, pregnancy, postpartum, menopause, and postmenopausal periods) with various women-related health conditions.
2. Intervention (I): WeChat-based nursing interventions (WeChat groups, official accounts, applets, and private chat) for health education, care management, psychological support, and multidisciplinary collaborative care.
3. Comparison (C): Standard routine care, usual hospital follow-up, or no additional WeChat-based intervention.
4. Outcomes (O): Clinical indicators, quality of life, self-management ability, treatment compliance, psychological status, satisfaction, and behavioral outcomes.

### Database Selection and Search Strategy

An extensive search plan was formulated by using free-text words and subject headings. The following databases were explored from January 2011 to December 2024: IEEE Xplore, Web of Science, PubMed, Scopus, ACM Digital Library, the Cochrane Central Register of Controlled Trials, and the China National Knowledge Infrastructure (CNKI). An effective retrieval strategy was established using a 2-step process. The first step involved collecting and organizing relevant search terms from related systematic reviews and meta-analyses [25,26]. Afterward, 3 authors (RL, YW, and TW) carefully examined and improved these terms to guarantee thorough inclusion of all essential terms. Using Boolean logic operators, the concluding search strategy was designed: (“wechat” or “weixin” or “official account” or “public account” or “applet” or “mini program”) and (medic* OR illness* OR disease* OR health* OR pharma* OR drug* OR therap*) and (woman OR women OR girl OR female). The full search strategy for each database is detailed in Table 1. Additionally, we did not impose any restrictions on health-related keywords in the Cochrane Central Register of Controlled Trials database, because we assumed that all documents in this database are related to human health by default, so that we can cover a wider range of potential literatures. Since numerous relevant studies have been published in Chinese, we specifically included CNKI. Other ways to identify studies involved expert recommendations and manually searching the references of included studies and pertinent systematic reviews.

**Table 1.** Specific search terms of databases.

Database	Search term	Studies, n
IEEE Xplore	((((All Metadata:“Wechat") OR (All Metadata:"weixin") OR (All Metadata:"official account") OR (All Metadata:"applet") OR (All Metadata:"Mini Program"))) AND ((All Metadata:medic*) OR (All Metadata:illness*) OR (All Metadata:disease*) OR (All Metadata:health*) OR (All Metadata:pharma*) OR (All Metadata:drug*) OR (All Metadata:therap*))) AND ((All Metadata:"woman") OR (All Metadata:"women") OR (All Metadata:"girl") OR (All Metadata:"female”))	6
Web of Science	((TS=(“Wechat” or “weixin” or “official account” or “applet” or “Mini Program”)) AND TS=(medic* OR illness* OR disease* OR health* OR pharma* OR drug* OR therap*)) AND TS=(“woman” or “women” or “girl” or “female”)	629
PubMed	((“Wechat” OR “weixin” OR “official account” OR “applet” OR “Mini Program”) AND (medic* OR illness* OR disease* OR health* OR pharma* OR drug* OR therap*)) AND (“woman” or “women” or “girl” or “female”)	575
ACM Digital Library	[[All: “wechat”] OR [All: “weixin”] OR [All: “official account”] OR [All: “applet”] OR [All: “mini program”]] AND [[All: medic*] OR [All: illness*] OR [All: disease*] OR [All: health*] OR [All: pharma*] OR [All: drug*] OR [All: therap*]] AND [[All: “woman”] OR [All: “women”] OR [All: “girl”] OR [All: “female”]]	583
Cochrane Central Register of Controlled Trials	“Wechat” or “weimin” or “official account” or “applet” or “Mini Program” in Title Abstract Keyword AND “woman” or “women” or “girl” or “female” in Title Abstract Keyword - (Word variations have been searched)	351
Scopus	TITLE-ABS-KEY (“Wechat” OR “weixin” OR “official account” OR “applet” OR “Mini Program”) AND TITLE-ABS-KEY (medic* OR illness* OR disease* OR health* OR pharma* OR drug* OR therap*) AND TITLE-ABS-KEY (“woman” OR “women” OR “girl” OR “female”)	900
China National Knowledge Infrastructure	(SU%='微信' OR SU%='公众号' OR SU%='小程序' OR SU%='服务号') AND (SU%='女性' OR SU%='妇女' OR SU%='女生' OR SU%='女童') AND (SU%='健康' OR SU%='医疗' OR SU%='疾病' OR SU%='卫生' OR SU%='护理' OR SU%='康复' OR SU%='健康教育' OR SU%='健康管理')	347

Retrieved records were first imported into EndNote X21. Duplicate records were identified using the automatic deduplication tool, and a manual check was conducted to remove any duplicates left due to differences in author names, publication formats, or metadata entries.

### Screening Process

The titles and abstracts were independently reviewed by two reviewers (JX and RL) following a step-by-step process using the established inclusion and exclusion criteria (Table 2). Any discrepancies were resolved through discussion until consensus; if unresolved, a third senior reviewer (TW) adjudicated the final decision. Cohen κ was used to assess the agreement between the reviewers.

**Table 2.** Inclusion and exclusion criteria for title and abstract screening.

Framework	Inclusion criteria	Exclusion criteria
Population	Women of all age groups and life stages with related health conditions	Non-female-specific diseases (asthma, appendicitis, etc)
Intervention	WeChat-based mHealth interventions in women (health education, symptom monitoring, care management, psychological intervention, etc) Focused on nursing-led or nursing-coordinated interventions, determined by the actual intervention content, the first/corresponding author’s nursing department affiliation, and author contribution statements	Investigated the relationship between WeChat use and other factors (mental health, etc) Computational analytical studies (content analysis, framework development, etc) Merely used WeChat for questionnaire distribution and data collection, with no active health intervention Irrelevant to WeChat-based mHealth interventions
Comparison	Standard care and follow-up	Not available
Outcomes	Clinical indicators, psychological status, self-management, compliance, and other health-related outcomes	Not available
Study design	Original research articles Interventional studies (randomized controlled trials and quasi-experiments) No language restrictions	Clinical trial protocols Noninterventional designs (cross-sectional, cohort, case series, etc) CNKI^a^-sourced literature outside Chinese core journal databases (Peking University Core, CSCD^b^, Chinese Science and Technology Core Journals)

a CNKI: China National Knowledge Infrastructure.

b CSCD: Chinese Science Citation Database.

### Quality Appraisal

The Cochrane risk-of-bias tool [27] and Risk of Bias in Nonrandomized Studies of Interventions (ROBINS-I) [28] were used to assess the quality of randomized controlled trials (RCTs) and quasi-experiments, respectively. Both instruments encourage assessment of internal and external validity and aid in evaluating methodological quality and clarity in reporting. Two independent reviewers (JX and RL) conducted the quality assessment for all studies. Any disagreements were resolved through discussion or by involving a third reviewer (TW).

### Data Preprocessing and Extraction

To support subsequent bibliometric analysis, records from all databases were standardized and manually adjusted to align with the Web of Science format. For studies with missing bibliometric information (eg, incomplete affiliation, citation counts, or journal metadata), supplementary information was manually retrieved from the original full text.

Before bibliometric analysis, data were systematically cleaned and standardized using the *bibliometrix* R packages. To ensure consistency, author names were standardized in terms of abbreviations, initials, and sequence. For example, different versions like “Zhang, San,” “Zhang, S.,” and “San Zhang” were unified into a consistent format. Synonyms, aliases, and different singular/plural versions of keywords were also unified. For example, “mini-program,” “mini program,” and “applet” were unified into a single term. Other core bibliographic information, such as first/corresponding authors, countries, affiliations, and journals, were also standardized. Ultimately, the cleaned bibliographic records were converted into a structured data frame, making them ready for further bibliometric analysis.

Given the diverse women’s health conditions, the small number of studies per condition, and clinical heterogeneity across included studies, a narrative descriptive synthesis was prespecified as the primary outcome analysis method. One reviewer (JX) independently extracted all data from each included study using the predefined form. Extracted items included author, publication year, country, study setting, study design, objectives, sample size, participant characteristics, intervention details, outcome measures, and key findings. A second reviewer (RL) independently verified all extracted data for accuracy and completeness. Discrepancies between the two reviewers (JX and RL) were resolved through discussion and consensus. If no agreement was reached, a third reviewer (TW) made the final decision. For missing study-level data, we only extracted and analyzed available valid data. No imputation or statistical filling was performed for missing data. The review adhered strictly to the registered protocol, with no deviations from the study plan.

### Bibliometric Metrics

VOSviewer (Leiden University) and InCites (Clarivate) were used to quantify the knowledge structure and emerging research trends. VOSviewer 1.6.18 was used to cluster institutions and researchers. To standardize terminological variations, a custom thesaurus file was used. Association strength normalization was applied, with attraction and repulsion values set at 3 and −1. Clustering was conducted using a resolution parameter of 1.0 and a minimum cluster size of 1, resulting in a network with 21 items, 103 links, and a total link strength of 106. The total citation impact was divided by the number of years since publication to determine the yearly citation impact. The H-index signifies that an author has H articles cited no less than H times.

## Results

### Process of Screening

**Figure 1.**
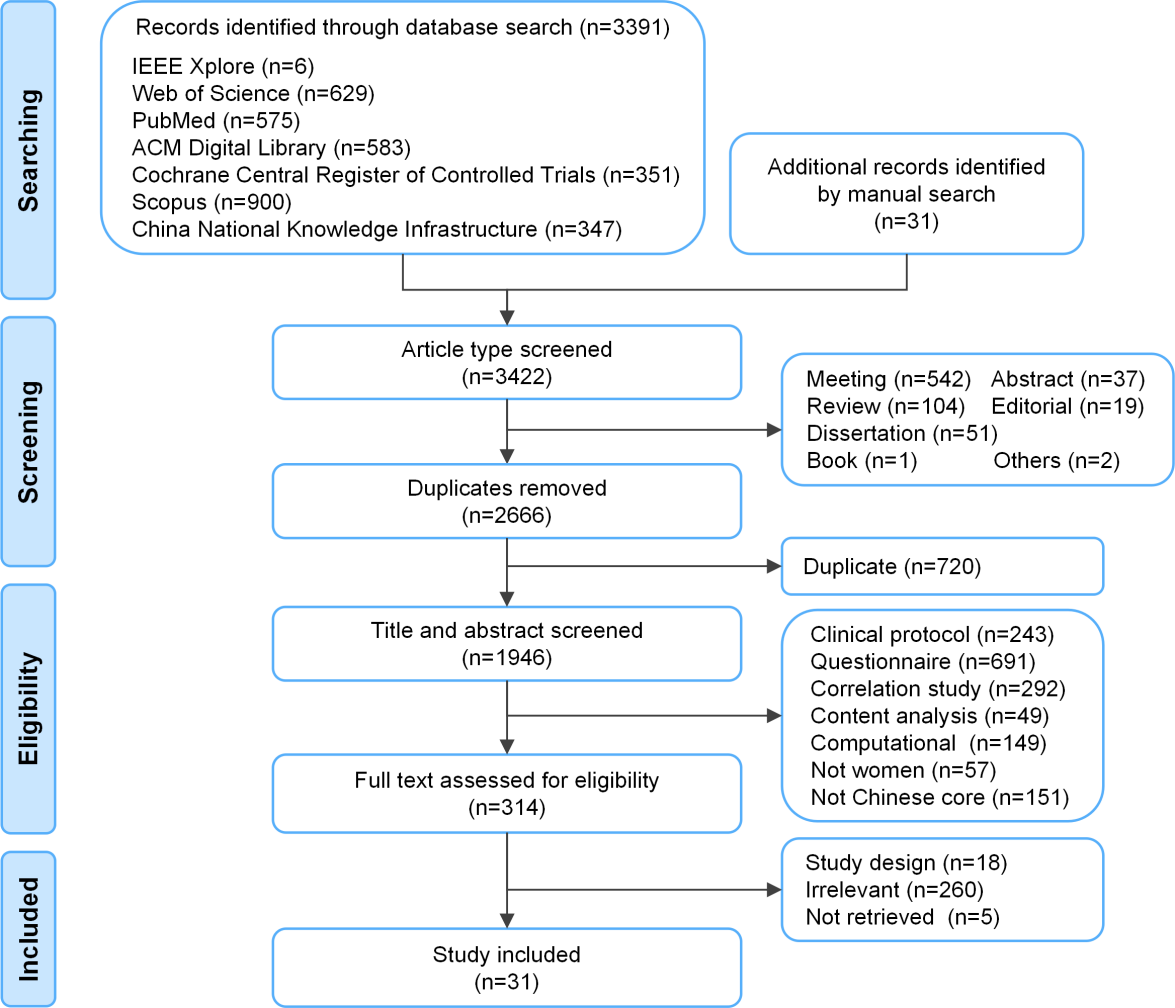
PRISMA (Preferred Reporting Items for Systematic Reviews and Meta-Analyses) flowchart.

Figure 1 presents a PRISMA flow diagram summarizing the screening process and exclusions. The electronic databases yielded 3391 records, and an extra 31 were found by manually searching journals, bringing the total initial number of records to 3422. After excluding nonoriginal articles and duplicates, the remaining 1946 records proceeded to title and abstract assessment. Of these, 1632 were excluded at this stage, and 314 full-text articles were assessed for eligibility. Finally, a set of 31 studies was included in the data extraction. Cohen κ was calculated to be 0.71, which suggests a substantial level of agreement.

### Basic Characteristics of Eligible Studies

The review systematically included 31 studies that appeared in publications between 2014 and 2024 (Table 3). Overall, 17 provinces participated in this research discourse, with the majority of publications originating from Beijing (n=5) [29-33], Shanghai (n=5) [34-38], Shanxi (n=3) [39-41], Liaoning (n=3) [42-44], and Zhejiang (n=3) [45-47]. Regarding the research type, 27 were RCTs and 4 were quasi-experiments [34,48-50], respectively.

**Table 3.** Study design, intervention modalities, and main outcomes of included studies.

Reference	Study design	Modality	WeChat intervention	Grouping and duration	Primary outcome
Prenatal care					
Guan et al [42] (2014)	RCT^a^	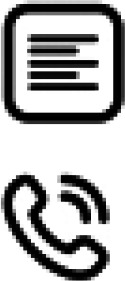	Establish a WeChat group and send prenatal examination times and prenatal education.	① Intervention (n=109) ② Usual care (n=109)	Higher rate of vaginal delivery, fewer negative emotions, and postpartum blood loss in ① than ②.
Huang et al [34] (2019)	QE^b^	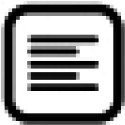	Establish an official account to push intervention information.	① Intervention (n=69) ② Usual care (n=69) *t*=13 weeks	Higher health belief in ① than ②. No significant behavior compliance and knowledge level.
Chen et al [48] (2020)	QE	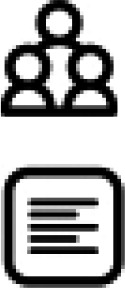	Share gestational knowledge, send course schedules, and push individualized prenatal care information.	① Intervention group (n=83) ② Regular examination (n=85) *t*=Until delivery	Higher number of prenatal examinations in ① than ②.
Gu et al [37] (2024)	RCT	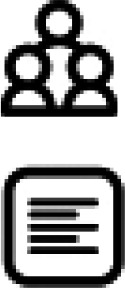	Make 8 appointments for midwife-led antenatal home visit services in the outpatient department and handling routine matters.	① Intervention (n=485) ② Usual care (n=499) *t*=Until delivery	Lower cesarean section rate, chances of epidural analgesia, and NICU^c^ admission, less amount of bleeding in the third stage of labor and 2 hours postpartum, and less amount of total bleeding in ① than ②.
Wang et al [51] (2024)	RCT	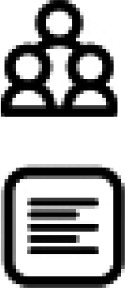	Based on the various stages of pregnancy, the intervention plan was formulated, incorporating motivation, ability, and trigger steps in each segment.	① Online and offline management (n=29) ② Standard care (n=48)	Reduced gestational weight gain, increased the appropriate weight gain rate, and improved perinatal outcomes in ① than ②.
Breast cancer					
Zhou et al [39] (2020)	RCT	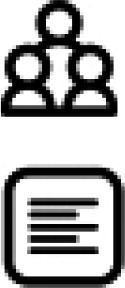	A multimodal nursing program offers guidance, education, and support on physical, mental, and social dimensions.	① Intervention (n=56) ② Routine care (n=55) *t*=6 months	Higher health-related quality of life during early rehabilitation in ① than ②. Comparable effects on pain, fatigue, and sleep disturbances in ① and ②.
Xu et al [52] (2021)	RCT	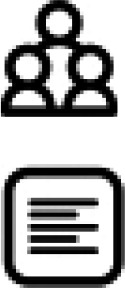	Establish a WeChat medical service team to create case management files and conduct health education.	① Intervention (n=63) ② Routine care (n=63)	Improved self-efficacy, quality of life, and satisfaction in ① than ②.
Xu et al [32] (2022)	Self-before-and-after	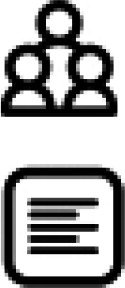	Join a WeChat group and explore the official account concerning complex decongestive therapy. The intensive phase spanned 1 month, and the maintenance phase was perpetual with weekly online instructions.	Received complex decongestive therapy with WeChat (n=61) *t*=3 months	Reduced excess arm volume, relieved lymphedema symptoms, and improved the quality of life after treatment.
Wu et al [47] (2021)	RCT	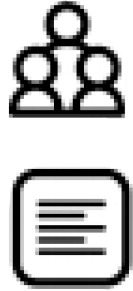	Group management and public account for education of patient’s family.	① Intervention (n=43) ② Usual care (n=43) *t*=3 months	Improved function of the affected limb, psychosocial adjustment ability, functional exercise compliance, quality of life, and nursing satisfaction in ① than ②.
Wang et al [38] (2024)	RCT	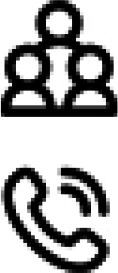	A nursing program with multiple modes delivers details on physical, psychological, and social rehabilitation.	① Intervention (n=62) ② Routine care (n=63) *t*=Until 6 months postsurgery	Reduced fear of cancer recurrence and increased health-related quality of life in ① than ②.
Gynecological cancer					
Zou et al [53] (2018)	RCT	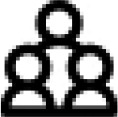	WeChat preoperative education includes psychological nursing, disease, HIFU,^d^ and the possible complications.	① Intervention (n=251) ② Usual care (n=175)	Reduced anxiety before and after surgery, decreased pain after surgery, and greater satisfaction with the treatment in ① than ②.
Lu et al [46] (2021)	RCT	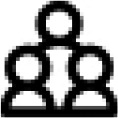	Establish an educational management group and provide targeted psychological intervention.	① Baofukang (n=50) ② Baofukang + Lactobacillus (n=50) ③ Baofukang + Lactobacillus + WeChat (n=50) *t*=3 months	Higher rate of lesion reversal, total effective rate, cognitive score in ③ than ① and ②.
Han et al [40] (2021)	RCT	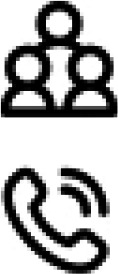	Multidisciplinary nursing collaboration is maintained continuously through WeChat, telephone, and face-to-face discussions.	① Intervention (n=66) ② Regular care (n=66) *t*=3 months	Attenuated anxiety and depression, higher physical functioning, general health, and mental health in ① than ②.
Li et al [41] (2023)	RCT	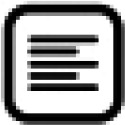	Deliver psychoeducation, skills training, and counseling through the official account “Hand in Hand.”	① WeChat couple-based psychosocial support (n=49) ② Eight WeChat articles (n=49) *t*=2 months	Higher relationship satisfaction and quality of life in ① than ②. Comparable effect on sexual function in ① and ②.
Tian et al [33] 2024	RCT	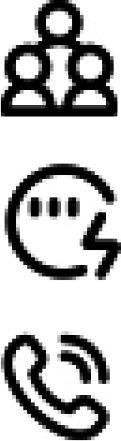	Join the nutrition management group on the “Good Nutrition” applet and send a private message for consultation.	① Intervention (n=48) ② Usual care (n=48)	Improved nutritional status in ① than ②. Significant altered nutrition-inflammation composite indices in ① and ②.
Gestational diabetes mellitus					
Tian et al [30] (2021)	RCT	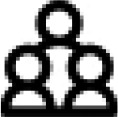	Patients share self-management experiences based on provided learning materials.	① Intervention (n=147) ② Standard care (n=162) *t*=Until delivery	More effective for blood glucose control in ① than ②. Pregnancy outcomes showed no significant differences.
Guo et al [54] (2021)	RCT	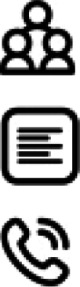	Establish a WeChat group, and push GDM^e^-related knowledge, conduct one-to-one guidance weekly and followed up online.	① Intervention (n=70) ② Routine care (n=70) *t*=Until delivery	Blood glucose levels, mental state, self-management behavior, quality of life, and pregnancy outcomes were better in ① compared to ②.
Deng et al [31] (2022)	RCT	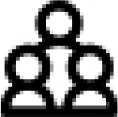	Remind participants to record diet and exercise diaries and post photos.	① Intervention (n=47) ② Routine care (n=47) *t*=Until delivery	Fewer GDM in ① than ②. Weight gain did not differ significantly.
Wang et al [49] (2024)	QE	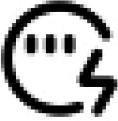	Pregnant women were guided offline on diet, exercise, etc, and used applet to upload data for online guidance.	① Intervention (n=26) ② Routine care (n=26) *t*=Until delivery	Reduced 2-hour postprandial glucose, improved self-management ability and blood glucose management satisfaction in ① than ②.
Breastfeeding					
Wu et al [29] (2020)	RCT	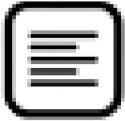	Push breastfeeding knowledge and promotion information through “Ke Xue Wei Yang.”	① Intervention (n=170) ② Routine care (n=174) *t*=Until 6 months postpartum	During the first month after birth, the rate of exclusive breastfeeding was greater in ① compared to ②.
Yu et al [55] (2021)	RCT	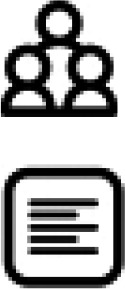	Deliver breastfeeding education and parenting insights, foster the mother-child relationship, and provide guidance on life specifics.	① Intervention (n=30) ② Usual care (n=30) *t*=3 months	The scores for breastfeeding efficacy and satisfaction, along with the exclusive breastfeeding rate, are superior in ① than in ②.
Polycystic ovary syndrome					
Sang et al [56] (2022)	RCT	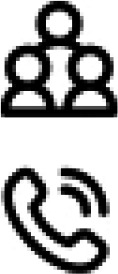	Patients report personal information and communicate with medical staff through WeChat.	① Intervention (n=39) ② Standard care (n=40) *t*=3 months	Improved weight loss and oocyte quality, and increased the live birth rate in ① than ②.
Dilimulati et al [36] (2024)	RCT	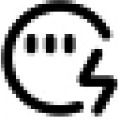	An applet with 3 modules: watching 2 videos about healthy lifestyle per week, recording health information, and self-selected reading. A doctor coached the patients weekly.	① Intervention (n=40) ② Metformin (n=40) *t*=3 months	Comparable insulin resistance in ① and ②. ① better than ② in reducing waist circumference, waist-to-hip ratio, total fat mass, and dehydroepiandrosterone sulfate. ① fewer than ② in adverse events.
Pregnant with psychological distress					
Yang et al [45] (2019)	RCT	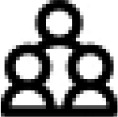	Join 4 mindfulness courses and a WeChat group to share experiences with researchers.	① Intervention (n=62) ② Routine care (n=61) *t*=2 months	Reduced depressive and anxious symptoms, as well as improved mindfulness skills in ① than ②.
Zhang et al [57] (2023)	RCT	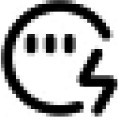	Reminder to participate in thematic lessons in video format and homework, such as mindful breathing and body scan practices.	① Intervention (n=80) ② Usual care (n=80) *t*=6 weeks	Reduced maternal depression and anxiety in ① than ②. More favorable temperament scores of infants in ① than ②.
Postpartum care					
Li et al [58] (2020)	RCT	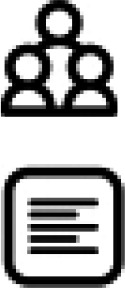	Push health education information via “maternal health care standard during pregnancy.”	① WeChat (n=350) ② Specialist team (n=350) ③ WeChat-specialist team (n=350) ④ Routine care (n=350) *t*=Until 7 weeks postpartum	Enhanced quality of maternal health care services in ①②③ than ④. ④ had the largest effect on both primary and secondary outcomes.
Chu et al [35] (2024)	RCT	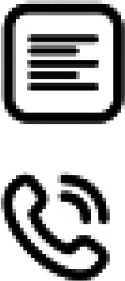	Deliver daily training reminders and see videos guiding pelvic floor muscle training.	① Intervention (n=76) ② Control (n=72) *t*=6 weeks	Increased adherence rate, improved peak surface electromyography, and contraction endurance in ① than ②. Comparable stress urinary incontinence symptoms in ① and ②.
Others					
Wang et al [43] (2021)	RCT (gynecological inflammatory disease)	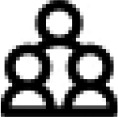		① Intervention (n=160) ② Usual care (n=160) *t*=3 months	The quality of nursing, understanding of gynecological health knowledge, and satisfaction level are higher in ① compared to ②.
Su et al [44] (2016)	RCT (primary dysmenorrhea)	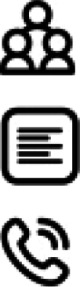		① Intervention (n=90) ② Routine care (n=90) *t*=3 months	Improved knowledge, attitude, behavior, and reduced pain intensity in ① than ②.
Li et al [59] (2017)	RCT (infertility)	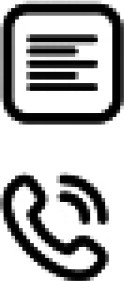		① Intervention (n=300) ② Usual care (n=300) *t*=3 months	Improved understanding of assisted reproduction, greater effectiveness in managing one’s own cycle, self-efficacy, and satisfaction, lower degree of anxiety in ① than ②.
Yu et al [50] (2023)	QE (pelvic organ prolapse)	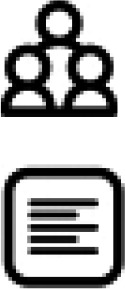		① Intervention (n=40) ② Routine care (n=40)	Improved cognition, compliance, pelvic floor function, muscle strength, and quality of life in ① than ②.

a RCT: randomized controlled trial.

b QE: quasi-experiment.

c NICU: neonatal intensive care unit.

d HIFU: high-intensity focused ultrasound.

e GDM: gestational diabetes mellitus.

### Intervention Method

**Figure 2.**
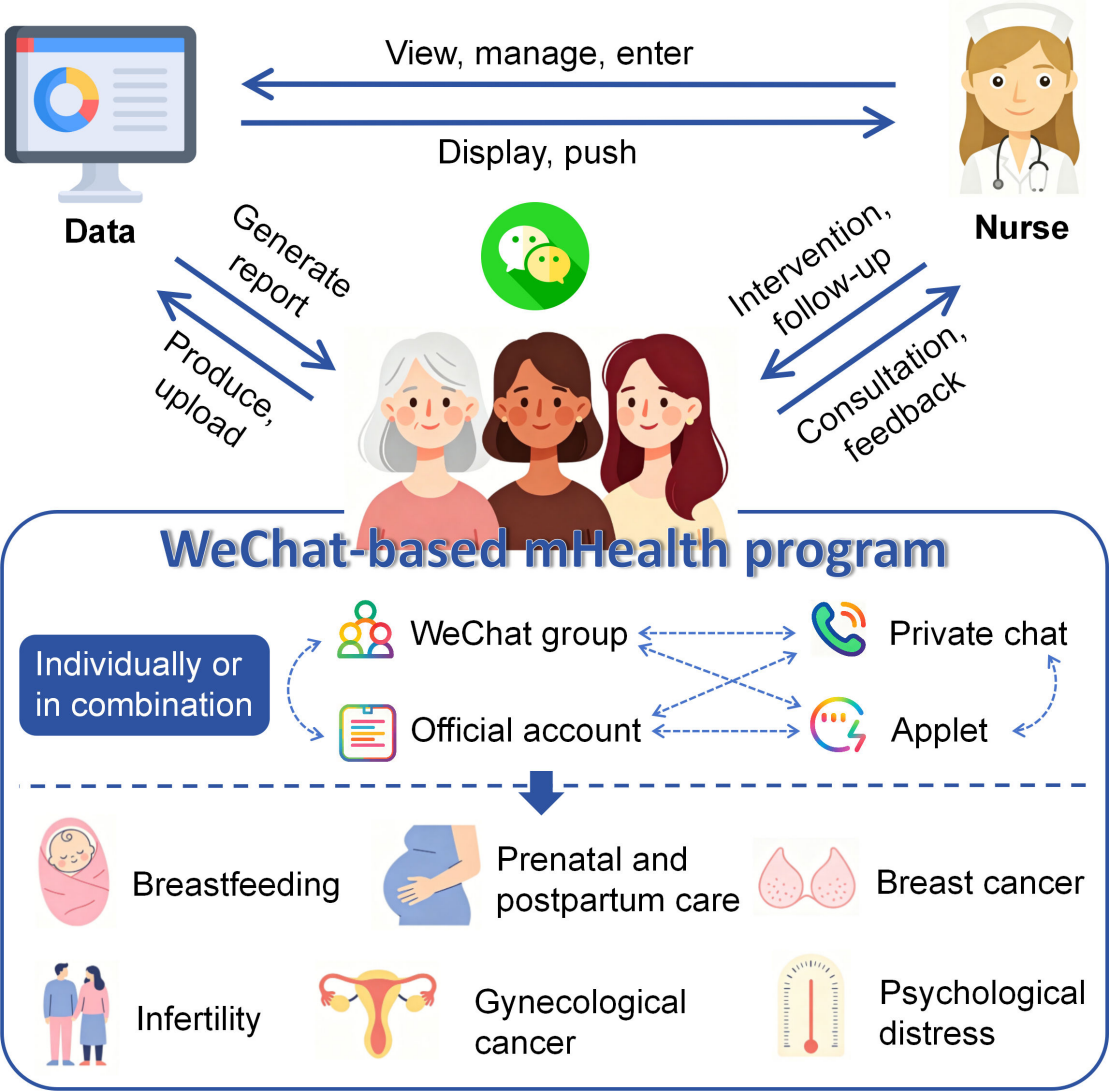
Conceptual logic of interventional studies, illustrating the full workflow, participant interaction patterns, and service components of WeChat-based nursing interventions. mHealth: mobile health.

In most studies, the control group received standard care, which included routine patient education and health management. For pregnant women, standard care typically involves regular classes at the maternity school and scheduled telephone follow-ups (Figure 2) [49]. The mHealth group comprises standard care plus WeChat interventions, which fall into 4 categories: WeChat group, official account, private chats, and applet. Twenty-two studies established WeChat groups, and participants could freely share experiences, discuss difficulties, and interact with peers and researchers at any time [30-33,37-40,43-48,50-56,58]. In these groups, all inquiries and consultations concerning the intervention were promptly handled by researchers. Nine studies used private chats for personalized follow-up communication, often in conjunction with group-based activities [33,35,38,40,42,44,54,56,59]. Eighteen studies delivered health-related information via official accounts to enhance the knowledge of diseases [29,32,34,35,37,39,41,42,44,47,48,50-52,54,55,58,59]. Some of these studies released original content, while others shared and reposted existing credible popular science articles from other sources. Four studies were developed to achieve more individual interaction [33,36,49,57]. An applet usually contains several modules, including a symptom assessment instrument and a health education knowledge base. They could also provide educational videos and check-in tasks [36]. Notably, 19 of the 31 studies used 2 or more intervention components in combination, indicating that combined WeChat-based strategies are common in mHealth interventions.

### Conditions Involved

The most studied diseases include prenatal care (n=5) [34,37,42,48,51], breast cancer (n=5) [32,38,39,47,52], and gynecological cancer (n=5) [33,40,41,46,53], followed by gestational diabetes mellitus (n=4) [30,31,49,54], breastfeeding (n=2) [29,55], polycystic ovary syndrome (n=2) [36,56], pregnant with psychological distress (n=2) [45,57], and postpartum care (n=2) [35,58]. There is only one study for gynecological inflammatory diseases [43], primary dysmenorrhea [44], pelvic organ prolapse [50], and infertility [59].

### Main Effectiveness of WeChat-Based Nursing Interventions

#### Prenatal Care

A total of 5 studies investigated WeChat-based interventions for prenatal care. Huang et al [34] reported no significant improvement in knowledge level or behavioral compliance despite increased health beliefs, whereas another two studies showed improvements in both knowledge and adherence [42,48]. Three studies found a lower cesarean section rate [37,42,51], and two of them reported reduced postpartum hemorrhage [37,42].

#### Breast Cancer

All 5 studies demonstrated that WeChat-based nursing interventions exerted favorable effects on psychological recovery and quality of life on women with breast cancer. Liang et al [32] exclusively assessed objective lymphedema-related outcomes and demonstrated clear relief of lymphedema. Wang et al [38] uniquely measured fear of cancer recurrence, which was significantly reduced in the intervention group. Xu et al [52] focused on self-efficacy and treatment-related discomfort, both of which showed significant benefits. Despite no studies reporting conflicting or opposite results, 2 studies consistently found that pain, fatigue, and sleep disturbance showed no statistically significant between-group differences [38,39].

#### Gestational Diabetes Mellitus

Altogether, 4 studies evaluated WeChat-based interventions for glycemic management in women with gestational diabetes mellitus [30,31,49,54]. All studies confirmed significant improvements in glycemic control (reduced fasting and 2-hour postprandial blood glucose levels, higher glycemic qualification rates), enhanced self-management ability, and lower incidence of adverse maternal and neonatal outcomes. Guo et al [54] specifically observed the improved psychological status after WeChat intervention. Tian et al [30] found that earlier intervention (23‐24 weeks) led to more stable glucose control.

#### Other Conditions

Two studies explored WeChat-based interventions to support breastfeeding. Both investigations yielded similar positive findings: elevated exclusive breastfeeding rates, strengthened breastfeeding self-efficacy, and boosted parental satisfaction relative to standard care [29,55]. However, Yu et al [55] centered on feeding safety in infants with congenital heart disease, while Wu et al [29] provided a longitudinal, population-level assessment of breastfeeding behaviors in healthy mother-infant dyads.

Two studies confirmed that digitally supported lifestyle modification significantly reduced body weight and BMI, and enhanced reproductive outcomes in patients with polycystic ovary syndrome relative to standard care [36,56]. These convergent results underscore the efficacy of WeChat platforms in facilitating sustainable lifestyle changes and clinical improvement.

Two studies revealed that digital mindfulness programs significantly alleviated depressive and anxiety symptoms and enhanced mindfulness capabilities [45,57]. Notably, the study of Zhang et al [57] was the first to reveal favorable ripple effects on infant neurodevelopment, with significantly better infant temperament at 6 weeks; these benefits were partially mediated by reduced pregnancy-specific anxiety.

In terms of postpartum rehabilitation, Chu et al [35] concentrated on postpartum pelvic floor muscle training, demonstrating that daily WeChat reminders more than doubled the adherence rate. By contrast, Li et al [58] evaluated comprehensive maternal health care quality, revealing that WeChat-based health education combined with specialist team support produced the highest satisfaction, followed by specialist support alone and WeChat alone, all superior to usual care.

### Multidisciplinary Participant

Six studies established multidisciplinary teams for mHealth initiatives [31,39,40,45,49,51]. In addition to physicians and nurses, midwives were typically included to support pregnant women. Psychologists or psychological counselors are incorporated in mindfulness interventions [45]. Within a diet and exercise program, nutritionists or dietitians would calculate the energy requirements for each pregnant woman, while exercise specialists provided tailored advice as necessary [31]. Pharmacists were tasked with developing medication plans for patients. To assess the quality of life in patients with cervical cancer, one study included a sex counselor [40]. When medication was required, researchers collaborated with endocrinologists to achieve optimal glucose control [49].

### Trend of Publication and Citation

The total number of publications shows a fluctuating trend. Prior to 2018, only a small number of articles were released annually [42,44,59]. From 2018 to 2024, there were at least 3 articles published each year, and the number of articles published was the highest in 2021 and 2024 (n=9 both). In terms of research fields, the majority of documents were categorized under obstetrics and gynecology (n=9), health care sciences services (n=7), general internal medicine (n=5), and medical informatics (n=5). Overall, this analysis emphasizes the interdisciplinary nature of the application of WeChat in women’s mHealth, which has attracted increasing interest and experienced substantial growth in recent years.

### Academic Output of Institutions

Analyzing institutional publications and citations offers a thorough insight into the scientific ecosystem. Altogether, 40 institutions participated in this field (Table 4). Multiple top-tier Chinese universities, including Peking University [32], Fudan University [37], Zhejiang University [45], and Shandong University [57], participated actively, alongside international institutions such as the University of Oxford [29], the National University of Singapore [38], and the University of Texas System [57]. Notably, Xi’an Jiaotong University distinguished itself as a productive contributor to the research with the highest citations (n=109) [39-41].

**Table 4.** List of organizations that published more than one paper.

Organization	Paper, n	Domestic document, n	International document, n	Times cited, n	Category Normalized Citation Impact
Shanghai Jiao Tong University	3	2	1	26	2.80
Xi’an Jiaotong University	3	2	1	109	3.19
Chinese Academy of Medical Sciences – Peking Union Medical College	3	3	0	56	1.55
Peking Union Medical College Hospital	3	3	0	56	1.55
Zhejiang University	2	2	0	71	2.99
Tongji University	2	2	0	16	2.29
Peking Union Medical College	2	2	0	53	1.98

### High-Impact Highly Cited Studies

Among the included studies, several publications emerged as highly cited and representative works in this field. Zhou et al [39] conducted the most frequently referenced study, which assessed a WeChat-based multimodal nursing program for women recovering from breast cancer surgery, and found notable enhancements in quality of life. Another work demonstrated that an 8-week WeChat-based mindfulness intervention effectively reduced anxiety and depression in pregnant women, and established a feasible model for psychological care via WeChat [45]. Wu et al [29] demonstrated that WeChat official account-based health education significantly improved exclusive breastfeeding rates. Tian et al [30] showed that WeChat group-mediated health education and lifestyle support yielded better glycemic control than standard prenatal care among women with gestational diabetes mellitus. Collectively, these highly cited studies represented key milestones and laid the methodological foundation for WeChat-based mHealth interventions in women’s health.

### Risk-of-Bias Assessment

**Figure 3.**
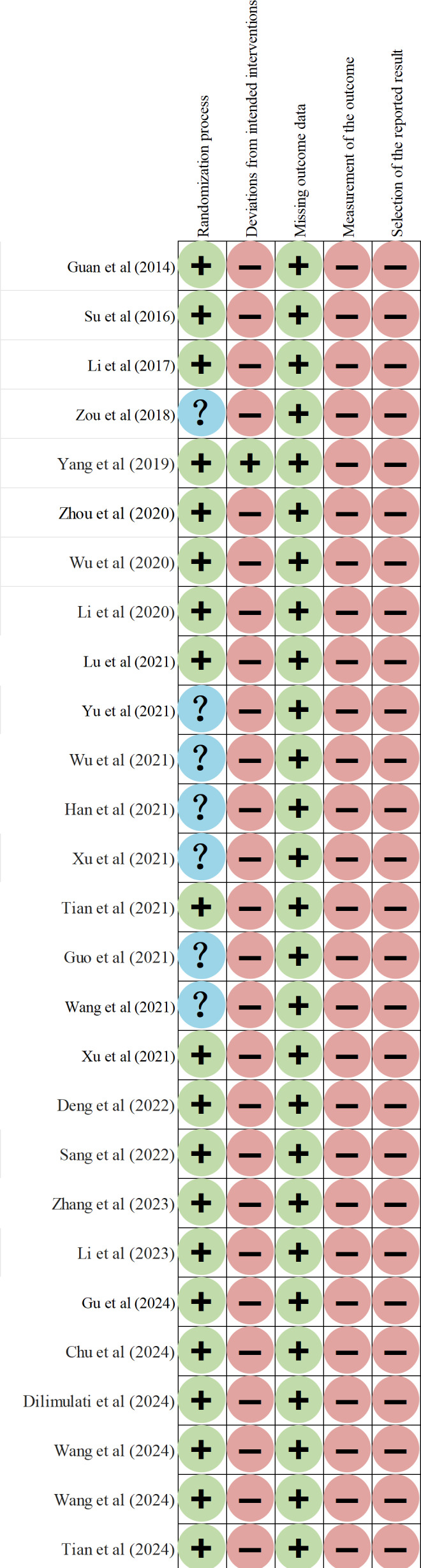
Risk of bias assessment by the revised Cochrane risk-of-bias tool for randomized trials “Rob2” [29-33,35-47,51-59].

In 20 studies, the randomization process was judged to be at low risk of bias, whereas 7 studies presented an unclear risk of bias in this domain (Figure 3 and Table 5) [40,43,47,52-55]. Blinding participants and personnel to group assignments was not feasible in all studies except one due to the interventions’ characteristics [45]. Lack of blinding may have altered participants’ health information-seeking behaviors in the control group, potentially biasing the outcome assessment. With regard to missing outcome data, all included studies were considered to have a low risk of bias. Regarding the measurement of outcomes and selection of the reported result, all studies were rated as having a high risk of bias, primarily because blinding of outcome assessors was not performed, and the reporting of outcomes was insufficiently transparent. Overall, the risk of bias across studies was predominantly high, which should be considered when interpreting the findings.

**Table 5.** Risk of bias in quasi-experiment studies with ROBINS-I^a^ tool.

Reference	Bias due to confounding	Bias in selection of patients into the study	Bias in classification of intervention	Bias due to deviations from intended interventions	Bias due to missing data	Bias in measurement of outcomes	Bias in selection of the reported result	Overall risk
Huang et al [34] (2019)	Serious	Low	Moderate	Moderate	Low	Low	Moderate	Serious
Chen et al [48] (2020)	Serious	Low	Moderate	Moderate	Low	Low	Moderate	Serious
Yu and Guo [50] (2023)	Serious	Low	Moderate	Moderate	Low	Low	Moderate	Serious
Wang et al [49] (2024)	Serious	Low	Moderate	Moderate	Low	Low	Moderate	Serious

a ROBINS-I: Risk of Bias in Nonrandomized Studies of Interventions.

## Discussion

To our knowledge, this is the first thorough review examining the impact of mHealth via WeChat on women. Although mHealth has been widely applied in various fields of obstetrics and gynecology, including prenatal care, postpartum health management, and gynecological disease prevention [60-62], the specific application of WeChat-based mHealth interventions in this population remains underexplored. In the included studies, WeChat-based mHealth was reported to be associated with greater access to personalized health information [63], observed to support self-management capabilities [64], and documented to enable real-time communication with health care providers [65]. This technology suggested beneficial effects on women’s proactive engagement in health monitoring and improving overall reproductive health outcomes.

As a popular social media platform in China, WeChat shows promising potential as an mHealth platform. It has been applied to support chronic disease management by providing personalized health guidance and support [63]. People do not need to download software specifically, and all functions are on WeChat, which people use every day. Specifically, WeChat’s group chat may help build health support groups and promote interaction and experience sharing among participants [33,66,67]. WeChat’s public account provides an effective channel for health education. Through public accounts, health professionals can regularly publish relevant information on health knowledge, disease prevention, and healthy lifestyles. This model was reported to reach large audiences and allow timely updates, with potential links to health literacy [68]. Finally, applets integrate health monitoring tools and personalized health plans, potentially supporting long-term health management and behavior tracking. This personalized intervention can provide customized health advice and support based on users’ specific health conditions and needs, thereby improving the effectiveness of health management [66]. Altogether, WeChat, through its diverse functions, provides strong support for the implementation of mHealth, becoming an important tool for promoting health management and health education.

Our findings revealed that the effective implementation of mHealth involved collaboration among multidisciplinary teams. Obstetricians play a central role in assessing and managing the health of pregnant women and adjusting treatment plans. Midwives provide support and care to ensure the safety and comfort of pregnant women [69]. In addition, the involvement of internal medicine physicians is crucial for managing chronic conditions in pregnant women, such as gestational diabetes or hypertension. Dietitians help pregnant women maintain a healthy weight and nutritional status through personalized dietary advice, thereby promoting the health of both mother and baby [31,51]. This multidisciplinary management model may improve the quality of obstetric care and strengthen communication and collaboration among health care providers [70].

The highly cited studies serve as pivotal clinical cornerstones for WeChat-based mHealth interventions in women’s health. These landmarks cover breast cancer, perinatal mental disorders, breastfeeding difficulties, and gestational diabetes mellitus. In most cases, these conditions demand continuous and personalized self-management, which conventional outpatient care struggles to fulfill efficiently. Breast cancer patients often face various physiological and psychological challenges during and after treatment, and remote rehabilitation effectively improves patients’ mental health and cognitive function [71]. Pregnant women are vulnerable to anxiety and depression and need timely psychological intervention [72]. Breastfeeding is one of the optimal ways for infants to obtain optimal nutrition; however, in practice, many mothers face various challenges. Therefore, sustained health education is crucial for the success of breastfeeding [73]. Meanwhile, women with gestational diabetes depend on regular glucose monitoring, dietary coaching, and behavioral supervision. Collectively, WeChat overcomes the temporal and spatial limitations of traditional care, enabling persistent, tailored, and responsive support.

Most eligible studies were RCTs, and RCTs are widely regarded as the gold standard in evidence-based medical research. Since mHealth interventions delivered via WeChat are difficult to blind in practice, performance bias and detection bias were almost unavoidable in the included trials. However, measurement bias and selective reporting bias are two common issues in these mHealth-related RCTs. Selective reporting bias suggests that researchers may selectively report certain outcomes while omitting or altering others to highlight statistically significant results. In leading journals in dentistry, the incidence of selective reporting bias is 51.5%, with the most common form being inconsistent assessment times for primary outcomes [74]. Even 85% of trials did not fully adhere to prespecified results in the antipsychotic drugs field [75]. Researchers and policymakers must enhance their understanding of the various assumptions, biases, and limitations to elevate the quality of RCTs. Moreover, RCTs ought to be combined with other research techniques to create a more thorough evidence base [76]. In particular, researchers should use objective, validated tools to measure key outcomes rather than relying solely on subjective self-reported data. For instance, researchers leveraged sensor data from smartphones and smartwatches combined with machine learning algorithms to detect emotional states and transitions [77]. By integrating deep neural networks with visual indicators, the mental health status of cancer patients can be objectively evaluated, achieving a high level of classification accuracy [78]. Only with improved methodological quality can this field establish a robust evidence base and achieve stable, scalable, and trustworthy mHealth models.

Our analysis has some limitations. First, current research mostly focuses on a single cultural context, while under multicultural settings, the effectiveness of WeChat mHealth apps may vary. Culturally adapted behavioral health interventions are more effective in improving health behaviors and outcomes [79]. The process of cultural adaptation is complex and requires multiple stages, including information collection, preliminary design, testing, refinement, and final implementation. Therefore, researchers can explore the adaptability and effectiveness of WeChat mHealth apps in different cultural contexts through cross-cultural comparative studies [80]. Second, despite many studies proving the short-term benefits of WeChat mHealth, conducting long-term follow-up studies can offer a deeper understanding of its enduring impact on health management and how it changes over different periods [81,82]. Third, combining WeChat with other technological platforms (such as wearable devices and electronic health record systems) could further enhance the effectiveness of these interventions [83].

WeChat‐based mHealth interventions show promising but still uncertain potential in improving access to personalized health information, enhancing self-management, and facilitating real-time doctor-patient communication. Given the high risk of bias across most included studies, the overall strength of evidence remains low and does not support definitive effectiveness. Multidisciplinary collaboration is crucial for effective mHealth implementation. Current limitations include a single-cultural context focus and a lack of long-term follow-up. Future studies should adopt more rigorous methodologies, including improved blinding, transparent outcome reporting, and validated objective measures. Cross-cultural adaptability, long-term effectiveness, and integration with wearable technologies should also be examined in subsequent research. This review fills a research gap and provides directions for optimizing WeChat-based mHealth interventions for women.

## Supplementary material

10.2196/82679 Checklist 1 PRISMA checklist.

## Abbreviations

CNKI: China National Knowledge Infrastructure
mHealth: mobile health
PICO: population, intervention, comparison, and outcomes framework
PRISMA: Preferred Reporting Items for Systematic Reviews and Meta-Analyses
RCT: randomized controlled trial
ROBINS-I: Risk of Bias in Nonrandomized Studies of Interventions
